# Role-reversals in Caregiving: Case Studies of Two Women Living With Late Stage Cancer

**DOI:** 10.1093/geroni/igab046.2927

**Published:** 2021-12-17

**Authors:** Candice Reel, J Hunter Williams, Emma Brennan, Jonna Williams, Kristen Payne, Rebecca Allen

**Affiliations:** 1 The University of Alabama, Tuscaloosa, Alabama, United States; 2 University of Alabama, Tuscaloosa, Alabama, United States

## Abstract

Many studies have examined the effects of caregiving burden and many others have focused on the effects of having a caregiver (Haynes-Lewis et al., 2018; Trevino, Prigerson, & Maciejewski, 2018; Semere et al., 2020). However, there is little data on the experience of role reversal, once responsible for caring for others and now being cared for while living with cancer. This project aims to identify ways in which women living with cancer cope with the internal struggles of receiving care. The current project is a case study of two females, one age 67, NHW, with a breast cancer diagnosis and one age 60, Black, with an ovarian cancer diagnosis, who once were caregivers and are now being cared for by family. Two semi-structured interviews were conducted that were approximately 60 minutes each. The study data are from a larger project focused on the self-perception of older women with late-stage cancer. Four independent researchers used thematic analysis to uncover common themes of coping between the two women receiving care. The themes uncovered were acceptance of the loss of autonomy, positive death attitudes, good relationships with their caregivers, and religiosity were identified and coded as coping strategies. The qualitative data showed that the use of these coping strategies helped the women be more accepting to care with less internal conflict. Future research should focus on generalizing these findings on a larger sample and use the data to help cancer patients better accept care from others.

